# Psychosis beas  a rare side effect of sildenafil: a case report

**DOI:** 10.1186/s13256-022-03334-6

**Published:** 2022-03-26

**Authors:** Mohammadreza Shalbafan, Maryam Orooji, Leila Kamalzadeh

**Affiliations:** 1 Mental Health Research Center, Psychosocial Health Research Institute, Department of Psychiatry, School of Medicine, Iran University of Medical Sciences, Tehran, Iran; 2 Department of Psychiatry, School of Medicine, Alborz University of Medical Sciences, Karaj, Iran

**Keywords:** Sildenafil citrate, Viagra, Erectile dysfunction, Psychotic disorders, Case report

## Abstract

**Background:**

Sildenafil citrate is a commonly used medication for the management of erectile dysfunction. Previous studies have described some neuropsychiatric side effects of this medication. So far, however, there has been little discussion about sildenafil-induced psychosis.

**Case presentation:**

We here present the case of a 32-year-old Iranian male, without a known psychiatric history, who developed psychotic symptoms following initiation of sildenafil. We also postulate a mechanism by which this may occur.

**Conclusions:**

This report highlights the importance of watchful observation for the occurrence of this rare but serious side effect. Further studies are needed to clarify the precise mechanism that causes sildenafil-induced psychosis.

## Background

Sildenafil citrate is a commonly used medication for the management of erectile dysfunction (ED). It amplifies the action of nitric oxide (NO) by selective inhibition of phosphodiesterase type 5 (PDE5), which is responsible for hydrolysis of cyclic guanosine monophosphate (cGMP) in the corpus cavernosum [1]. Accumulation of intracellular cGMP results in relaxation of penile smooth muscles, dilatation of arterioles, and increased blood flow, causing penile erection [2]. Although sildenafil appears to be very effective in the treatment of ED, some concerns regarding its side effects need to be addressed. Commonly reported side effects of sildenafil include headache, skin flushing, indigestion, and visual disturbances [3]. Neuropsychiatric adverse effects, including lightheadedness, depression, anxiety, sleeplessness, abnormal dreams, behavior changes, and nervousness, have also been reported in previous studies [4]. So far, however, there has been little discussion about sildenafil-induced psychosis. Although rare, this serious side effect results in negative outcomes, even in the presence of favorable medication response. Hence, physicians should be mindful of the possibility of this side effect. Here we present a patient, without a known psychiatric history, who developed psychotic symptoms following initiation of sildenafil. We also postulate a mechanism by which this may occur. This report highlights the importance of watchful observation for the occurrence of rare but serious side effects of risperidone.

## Case presentation

A 32-year-old Iranian male was referred to our hospital owing to psychotic symptoms including delusional jealousy, delusion of reference, and auditory hallucinations. The psychotic symptoms had become apparent about 1 week before his referral, when his family noticed that he sometimes talked to himself and became very hostile toward them. He also suffered from insomnia and irritability and had outbursts of aggressive behavior. There were no symptoms consistent with mania or hypomania, and he did not have any previous episodes of psychotic or mood symptoms. The patient did not report a history of exposure to toxic chemicals, head trauma, seizures, or other medical conditions that could cause psychosis. He was recently diagnosed with mild erectile dysfunction (International Index of Erectile Function score 20) [5] and had been prescribed sildenafil 50 mg as a single dose, no more than once a day, 1 hour before sexual intercourse over the past 2 weeks. He did not use any other medications while using sildenafil. He smoked tobacco for the past 4 years and did not have a history of alcohol or illicit drug abuse. There was no family history of psychiatric disorders. Birth and early development were apparently within normal limits. School achievements were average, and since graduation from high school, the patient has worked in a factory. His family described him as an extroverted, hardworking, and responsible person. On mental status examination, he was alert and oriented to place, time, and person. His physical examination, including a thorough neurological and genitourinary assessment, was unremarkable. All routine blood tests, including complete blood count (CBC), fasting blood glucose, hemoglobin A1c, lipid profile, electrolytes, renal and liver function tests, thyroid function tests, vitamin B12, folic acid, and vitamin D levels, and an electrocardiogram were within normal limits. Tests for syphilis, human immunodeficiency virus (HIV), and hepatitis B and C as well as toxicology screening were negative (Table 1).

**Table 1:** Laboratory investigations performed on admission

Complete blood count	WBC: 6.68 × 10^9^/L
	RBC: 5.43 × 10^12^/L
	Hemoglobin: 15 g/dL
	Hematocrit: 39.8%
	Mean corpuscular volume (MCV): 91.9 fL
	Platelets: 350 × 10^9^/L
Erythrocyte sedimentation rate (ESR)	18 mm/hour
Fasting plasma glucose	90 mg/dL
Hemoglobin A1c	6%
Cholesterol, total	157 mg/dL
Cholesterol, LDL	92 mg/dL
Cholesterol, HDL	61 mg/dL
Triglycerides	148 mg/dL
Sodium (Na)	142 mmol/L
Potassium (K)	4.3 mmol/L
Calcium (Ca)	8.6 mg/dL
Magnesium (Mg)	2 mg/dL
Urea (BUN)	16 mg/dL
Creatinine	0.9 mg/dL
Alanine aminotransferase (ALT)	34 units/L
Aspartate aminotransferase (AST)	32 units/L
Alkaline phosphatase	66 units/L
Albumin	4.1 g/dL
Bilirubin—direct	0.2 mg/dL
Bilirubin—total	0.4 mg/dL
Thyroid-stimulating hormone (TSH)	2.5 μIU/mL
Thyroxine (T4)—free	7 μg/dL
Triiodothyronine (T3)—total	135 ng/dL
Uric acid	4.5 mg/dL
Vitamin B12	653 pg/mL
1,25-Dihydroxycholecalciferol (calcitriol)	54 pg/mL
Folate (folic acid)	16 ng/mL
Urine toxicology screening	Opiates: negative
	Cannabinoids: negative
	Methadone: negative
	Amphetamines: negative
	Methamphetamine: negative

*ALT* Alanine aminotransferase, *AST* Aspartate aminotransferase, *BUN* Blood urea nitrogen, *ESR* Erythrocyte sedimentation rate, *HDL* High-density lipoproteins, *LDL* Low-density lipoproteins, *MCV* Mean corpuscular volume, *RBC* Red blood cells, *TSH* Thyroid-stimulating hormone, *T3* Triiodothyronine, *T4* Thyroxine, *WBC* White blood cells

Magnetic resonance imaging was performed to rule out space-occupying lesions or vascular infarcts. Given his state, the patient was admitted to the psychiatric ward. Sildenafil was discontinued, and oral form of risperidone was prescribed at 4 mg/day. Surprisingly, all of the psychotic symptoms remitted only 1 week after the initiation of risperidone, and subsequently he was discharged after 10 days of hospitalization. On the basis of the clinical presentation, investigations, and the treatment outcome, a provisional diagnosis of sildenafil-induced psychotic disorder according to the Diagnostic and Statistical Manual of Mental Disorders, Fifth Edition (DSM-5) criteria was considered. One month after hospital discharge, the patient reported no psychiatric symptoms and risperidone was tapered off in 2 weeks. Approximately 4 months later, he was brought to clinic by his family because of recurrence of similar psychotic symptoms that occurred after taking sildenafil only once. Risperidone was started again and gradually titrated up to 4 mg/day. The patient recovered completely in 2 weeks. Follow-up visits during the next 12 months revealed no recurrence of the symptoms.

## Discussion and conclusions

Neuropsychiatric complications of medications have always been important and challenging issues that interfere with diagnosis and management of patients [6]. In this paper, we report a psychotic episode associated with the use of sildenafil in a subject without personal or family history of mental illness. Although different neuropsychiatric side effects of sildenafil such as aggressive behavior, depression, abnormal dreams and nervousness, rape, and suicidal attempt have been published so far [7], literature about sildenafil-induced psychosis is scanty. In 2004, Baggot and Singh reported a relapse of mixed affective state with paranoid delusions and elevated mood in a bipolar patient treated with sildenafil. In this patient, affective symptoms improved with discontinuation of sildenafil and prescription of antipsychotics. The authors focused on the role of NO in the pathophysiology of psychotic disorders and hypothesized that an excess of NO-stimulated cGMP due to the inhibition of PDE led directly to the relapse [8]. In the same vein, Özdemiroğlu *et al*. reported on a patient who first developed mania while taking pramipexole, and some additional psychotic symptoms further emerged when panax ginseng and sildenafil were added to the ongoing pramipexole treatment. Although a link between sildenafil and psychosis has been suggested in this article, concomitant medications such as ginseng and pramipexole were other possible causes of psychotic symptoms. Besides, the patient had Parkinson’s disease, which is associated with a predisposition to psychosis [9]. In terms of pathophysiology, there is some evidence available to suggest a possible association between sildenafil and psychotic symptoms. As mentioned earlier, the main pharmacological action of sildenafil is the inhibition of the cGMP-specific PDE5. PDE5 is present in substantial concentrations in the smooth muscles of the systemic vasculature and cerebral neurons and vessels [10]. Inhibition of cGMP degradation by selective PDE5 raises nitric oxide (NO) levels by increasing the ratio of nitrite to nitrate and by stimulating transcription of mRNA for nitric oxide synthase (NOS) [11]. NO is a key component in many processes occurring in the nervous system such as regulation of synaptic plasticity [12], neurotransmission [13], and development of nervous tissue [14]. However, nitric oxide can be considered a double-edged blade. In fact, in the low, regulated mode, NO has favorable effects, mediating and protecting neuronal function. On the other hand, in the high, unregulated mode, it has neurotoxic effects [15]. A growing body of evidence suggests that NO is involved in cerebrovascular diseases, seizures, neurodegenerative disorders, and pain [16]. Previous studies have also concentrated on the association between NO and psychotic disorders (17, 18). It is not unlikely that sildenafil’s inhibition of PDE5, accumulation of cGMP, and alterations in the concentrations of NO in the brain would result in psychotic symptoms. Accordingly, it is plausible that other PDE5 inhibitors such as tadalafil, vardenafil, and avanafil would be able to cause such symptoms through a similar mechanism. Nonetheless, in reviewing the literature, we found no reports on other PDE5 inhibitors causing psychotic symptoms. There are, therefore, other possible mechanisms behind the central nervous system adverse effects of the sildenafil that until now have not been recognized. In our case, there was a close temporal relationship between the use of sildenafil and emergence of psychotic symptoms. Furthermore, discontinuation of the sildenafil led to quick and full recovery from psychotic symptoms. These data strongly support a causative role for sildenafil in the development of psychotic symptoms. Taken together, this case demonstrates an episode of psychosis arising as a possible side effect of sildenafil and highlights the importance of watchful observation for the occurrence of this rare but serious side effect. Further studies are needed to clarify the precise mechanism that causes sildenafil-induced psychosis.

## Data Availability

Data sharing is not applicable to this article as no datasets were generated or analyzed during the current study.
